# Chip-integrated quantum signature network over 200 km

**DOI:** 10.1038/s41377-025-01775-4

**Published:** 2025-03-04

**Authors:** Yongqiang Du, Bing-Hong Li, Xin Hua, Xiao-Yu Cao, Zhengeng Zhao, Feng Xie, Zhenrong Zhang, Hua-Lei Yin, Xi Xiao, Kejin Wei

**Affiliations:** 1 Guangxi Key Laboratory for Relativistic Astrophysics, School of Physical Science and Technology, Guangxi University, Nanning, 530004 China; 2 National Laboratory of Solid State Microstructures and School of Physics, Collaborative Innovation Center of Advanced Microstructures, Nanjing University, Nanjing, 210093 China; 3 National Information Optoelectronics Innovation Center (NOEIC), Wuhan, 430074 China; 4 Guangxi Key Laboratory of Multimedia Communications and Network Technology, School of Computer, Electronics, and Information, Guangxi University, Nanning, 530004 China; 5 Department of Physics and Beijing Key Laboratory of Opto-electronic Functional Materials and Micro-nano Devices, Key Laboratory of Quantum State Construction and Manipulation (Ministry of Education), Renmin University of China, Beijing, 100872 China; 6 Peng Cheng Laboratory, Shenzhen, 518055 China

**Keywords:** Quantum optics, Integrated optics

## Abstract

The development of quantum networks is paramount towards practical and secure communications. Quantum digital signatures (QDS) offer an information-theoretically secure solution for ensuring data integrity, authenticity, and non-repudiation, rapidly growing from proof-of-concept to robust demonstrations. However, previous QDS systems relied on expensive and bulky optical equipment, limiting large-scale deployment and reconfigurable networking construction. Here, we introduce and verify a chip-based QDS network, placing the complicated and expensive measurement devices in the central relay while each user needs only a low-cost transmitter. We demonstrate the network with a three-node setup using an integrated encoder chip and decoder chip. By developing a 1-decoy-state one-time universal hashing-QDS protocol, we achieve a maximum signature rate of 0.0414 times per second for a 1 Mbit messages over fiber distances up to 200 km, surpassing all current state-of-the-art QDS experiments. This study validates the feasibility of chip-based QDS, paving the way for large-scale deployment and integration with existing fiber infrastructure.

## Abstract (web summary)

A novel chip-based QDS network, utilizing an advanced protocol, achieves a signing rate of 0.04 times per second over 200 km for 1-Mbit messages, representing a significant advancement in QDS.

## Graphical abstract

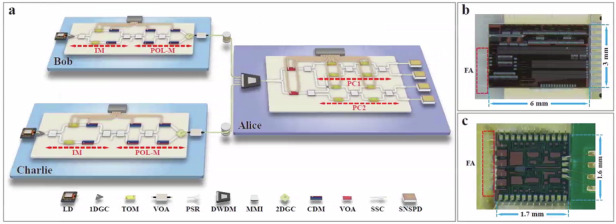

## Introduction

Cryptography is widespread in modern society and crucial for numerous applications, including e-commerce, digital currencies, and blockchain, all of which depend on data confidentiality, integrity, authenticity, and non-repudiation. Currently, these applications’ security relies heavily on public-key cryptography^1,2^, which is believed to be secure against eavesdroppers with limited computational capabilities. However, the security of this cryptographic approach is at risk due to rapid developments in algorithms^3,4^ and computational power, particularly in the field of quantum computing^5–8^.

Unlike classical cryptography, quantum cryptography, utilizing quantum mechanical properties^9^, provides a cryptographic toolbox without relying on any assumptions about the computational power of eavesdroppers. A well-known example of quantum cryptography is quantum key distribution (QKD), which offers an information-theoretically secure encryption solution to the key sharing problem, making assumptions only about the devices owned by authorized users^10,11^. With much efforts, QKD has achieved significant milestones, reaching distances of up to 1000 km^12^ and integration into backbone fiber infrastructure of classical communications^13,14^.

Different from QKD, quantum digital signatures (QDS) enables users to sign documents using quantum methods so that they can be transferred with information-theoretic integrity, authenticity, and non-repudiation. This plays a crucial role in emails, software distribution, and financial transactions, where data integrity against forgery is paramount. The first QDS protocol was proposed in 2001^15^, but it was impractical due to the need for long-term quantum storage and secure quantum channels. Substantial efforts have eliminated these impractical technical requirements^16–19^, and the practical performance of QDS regarding security and signature efficiency has been significantly enhanced^20–22^. Specifically, a novel scheme named the one-time universal hashing (OTUH)-QDS protocol, first proposed by Yin et al.^23^ and further developed by Li et al.^24^, significantly boosts signature efficiency by enabling users to sign messages of any length with information-theoretic security.

In experiments, QDS has developed from proof-of-principle demonstrations to long distances^25–27^, GHz repetition rates^28–30^, field tests^31–33^, and reconfigurable networks^34–36^. These achievements bring QDS closer to maturity and are believed to be the next step in commercial quantum technologies^37^. However, all previous works rely entirely on bulky and expensive optical setups, encountering significant challenges for wide deployments and easy integration of QDS with existing backbone fiber infrastructures.

In this work, we introduce and verify a chip-based QDS network. In such a network, each user requires only an integrated photonic transmitter chip, while the complex and expensive measurement devices are placed in the central node. We further address the technical challenges of building such a network by developing the 1-decoy-state OTUH-QDS protocol, which allows efficient signatures using one decoy state and non-privacy-amplification keys. This dramatically reduces the manufacturing complexity of the transmitter chip and reduces the computational cost and latency of the post-processing stage. We demonstrate the network with a three-node setup that achieves a maximum signature rate of 0.0414 times per second (tps) for 1 Mbit messages over fiber distances up to 200 km. This signature rate surpasses current state-of-the-art QDS experiments. This study validates the feasibility of chip-based QDS, paving the way for large-scale deployment and integration with existing fiber infrastructures.

## Results

### Network structure

**Fig. 1:**
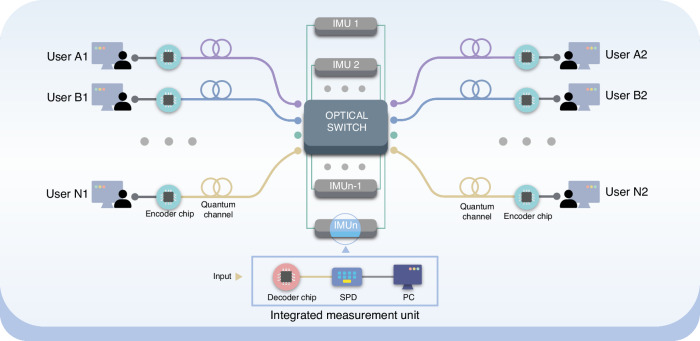
Schematic of the star-like topology chip-based QDS network. It primarily consists of three parts: end-users, optical switch, and integrated measurement units (IMUs). Each user holds a transmitting chip to produce quantum states, connected to the optical switch via fiber links. The optical switch can group users arbitrarily and route the transmitted quantum states to a specified IMU. The IMU mainly comprises quantum state decoding chip, single-photon detectors (SPDs), and personal computer (PC). This network architecture allows for reconfiguring IMU to run BB84 or MDI QDS protocol

The schematic of our proposed QDS network is shown in Fig. 1. The network features a star-like topology and consists of three main components: end-users, optical switches, and integrated measurement units (IMUs). Each user at the terminal nodes of the network has a compact transmitter chip and is linked to a central node containing several IMUs, including quantum state decoding chips, single-photon detectors (SPDs) and personal computers (PCs). To implement the QDS task, which typically involves three parties (namely a signer, a verifier, and a receiver), an optical switch or a dense wavelength division multiplexer (DWDM) is used to arbitrarily group two users as a verifier and a receiver and route the transmitted quantum states to any available IMU, assigned to a signer.

The network allows additional users to join by using time-division multiplexing technology. For example, an *N* × 1 optical switch enables *N* users to share the same IMU through time-division multiplexing. When the number of users reaches the capacity of one IMU, a mesh network structure^38^, using adjacent optical switches, accommodates these users by adding more IMUs, thereby further extending the network.

This architecture offers three main advantages. Firstly, each user only needs a compact transmitter chip fabricated by integrated platforms. Integrated photonics provides highly robust manufacturing processes that help reduce costs for personal devices and enable the miniaturization of components and circuits for handheld and field-deployable devices. Secondly, the signer holds the expensive and bulky measurement system, which is shared by all terminal node users, thus bypassing the challenging technique of integrating SPDs on a chip^39,40^, as users do not need to perform quantum detection. Thirdly, this network architecture easily integrates into existing classical telecommunications infrastructure and is compatible with the current quantum network by flexibly configuring the IMU^41–44^. For example, the network can upgrade to a measurement-device-independent version by using a Bell-state measurement device^34,45–47^.

### Efficient QDS protocol

In order to enhance the performance and compatibility with chip-based network structures, we develop a modified QDS protocol described in refs. ^23,24^. By employing a one-time universal hash function to produce a fixed-length digest representing the document’s characteristics and developing an advanced security proof, the protocol is capable of signing an arbitrarily long document with imperfect pre-distribution keys. Our main modification is using 1-decoy-state method in the pre-distribution state. This modification is crucial for building a practical QDS network:

Firstly, as this protocol does not require the preparation of a vacuum state, it lowers the extinction ratio requirement for intensity modulator (IM) on a silicon-based chip, thereby decreasing the manufacturing complexity of the transmitter chip. Secondly, as this protocol requires fewer resources for the generation and management of decoy states, it further reduces system complexity. Thirdly, due to its ability to directly utilize imperfect keys without privacy amplification, our protocol can significantly reduce the computational costs and latency associated with post-processing.

**Fig. 2:**
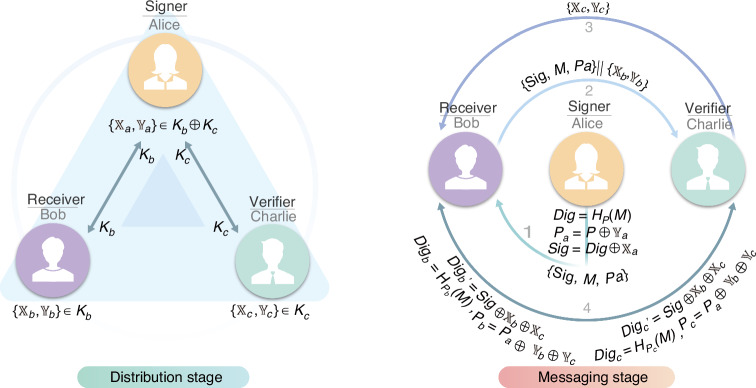
Schematic diagram for the efficient QDS Protocol. This protocol can be divided into two stages, known as distribution state and messaging state

Here, we summarize the main steps of our protocol. Details of the specific implementation and the security proof can be found in Supplementary Information Section 1 and Section 2. Our protocol, depicted in Fig. 2, is divided into two stages: distribution and messaging stage, outlined as follows.

#### Distribution stage

Bob (Charlie) and Alice share a sequence of raw keys by implementing the 1-decoy-state BB84 key generation protocol (KGP) to create a key bit string *K*_*b*_ (*K*_*c*_) of length *n*_*Z*_. That is, Bob (Charlie) sends a signal state with average intensity *μ* and probability *P*_*μ*_, and a decoy state with average intensity *ν* and probability *P*_*ν*_. Alice then measures them using the basis {*Z*, *X*} and generates *K*_*b*_ (*K*_*c*_) with an error correction algorithm. Note that *K*_*b*_ and *K*_*c*_ are not required for the privacy-amplification process; that is, all key bits are non-privacy-amplification key bits.

Alice subsequently creates a key string *K*_*a*_ by performing an XOR operation on the key strings *K*_*b*_ and *K*_*c*_, i.e., *K*_*a*_ = *K*_*b*_ ⊕ *K*_*c*_. To sign a message *M*, Alice randomly selects 2*L* bits from *K*_*a*_ to create two *L*-bit key strings $\left\{{{\mathbb{X}}}_{a},{{\mathbb{Y}}}_{a}\right\}$ and shares the positions of these bits with Bob (Charlie) via an authenticated channel. Bob and Charlie then independently extract their key strings $\left\{{{\mathbb{X}}}_{b},{{\mathbb{Y}}}_{b}\right\}$ and $\left\{{{\mathbb{X}}}_{c},{{\mathbb{Y}}}_{c}\right\}$ from *K*_*b*_ and *K*_*c*_ based on the positions specified by Alice and ensure that the relationships ${{\mathbb{X}}}_{a}={{\mathbb{X}}}_{b}\oplus {{\mathbb{X}}}_{c}$ and ${{\mathbb{Y}}}_{a}={{\mathbb{Y}}}_{b}\oplus {{\mathbb{Y}}}_{c}$ are satisfied. The three parties then initiate the messaging stage. Here, *L* is determined by preset system security parameters, $\epsilon =\max \{{\epsilon }_{{\rm{rob}}},{\epsilon }_{{\rm{rep}}},{\epsilon }_{{\rm{for}}}\}$. The security parameter *ϵ* is defined as the maximum probability of the QDS protocol failing, i.e., the probability that an attacker successfully forges, repudiates, or tampers the signature.

#### Messaging stage

Alice creates an *L*-bit digest *Dig* for the message *M* using a generalized division hash operation *Dig* = *H*_*P*_(*M*), characterized by a local random sequence *P*. She then encrypts *Dig* and *P* using key strings ${{\mathbb{X}}}_{a}$ and ${{\mathbb{Y}}}_{a}$, obtaining ${P}_{a}=P\oplus {{\mathbb{Y}}}_{a}$ and the signature $Sig=Dig\oplus {{\mathbb{X}}}_{a}$. Alice transmits {*Sig*, *M*, *P*_*a*_} to Bob via an authenticated channel.

Upon receiving the string, Bob forwards {*Sig*, *M*, *P*_*a*_} along with $\left\{{{\mathbb{X}}}_{b},{{\mathbb{Y}}}_{b}\right\}$ to Charlie. Subsequently, Charlie also transfers $\left\{{{\mathbb{X}}}_{c},{{\mathbb{Y}}}_{c}\right\}$ to Bob. Bob (Charlie) then independently generates an expected digest $Di{g}_{b}^{{\prime} }=Sig\oplus {{\mathbb{X}}}_{b}\oplus {{\mathbb{X}}}_{c}$ ($Di{g}_{c}^{{\prime} }=Sig\oplus {{\mathbb{X}}}_{b}\oplus {{\mathbb{X}}}_{c}$) and an actual digest $Di{g}_{b}={H}_{{P}_{b}}(M)$ ($Di{g}_{c}={H}_{{P}_{c}}(M)$), where ${P}_{b}={P}_{a}\oplus {{\mathbb{Y}}}_{b}\oplus {{\mathbb{Y}}}_{c}$ (${P}_{c}={P}_{a}\oplus {{\mathbb{Y}}}_{b}\oplus {{\mathbb{Y}}}_{c}$). They then verify the digests. If $Di{g}_{b}^{{\prime} }=Di{g}_{b}$ and $Di{g}_{c}^{{\prime} }=Di{g}_{c}$, the signature is accepted; otherwise, it is rejected.

Considering the security framework of a one-time universal hash^23,24^ and the 1-decoy-state BB84 KGP^48^, we can define the achievable signature rate as

1 $${R}_{S}=\frac{{n}_{Z}}{2Lt}$$

Here, *t* represents the cumulative time required to obtain a raw key of length *n*_*Z*_.

### Experimental setup

**Fig. 3:**
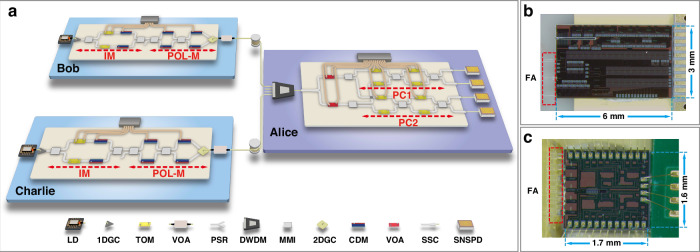
Experimental setup. **a** Experimental setup of chip-based three-node quantum network. It mainly consists of two quantum links: Bob-Alice and Charlie-Alice, connected by spooled fibers. The signal lights from Bob and Charlie are multiplexed by a dense wavelength division multiplexer (DWDM) before being measured by Alice. Bob and Charlie each have a laser diode (LD), a silicon-based encoder chip, and an off-chip variable optical attenuator (VOA). Alice has a DWDM, a silicon-based decoder chip with polarization tracking capability, and four superconducting nanowire single-photon detectors (SNSPDs). The encoder chip includes a one-dimensional grating coupler (1DGC) and a two-dimensional grating coupler (2DGC) for optical pulse input and output. The intensity modulator (IM) and polarization modulator (Pol-M) consist of multimode interferometers (MMIs), thermo-optical modulators (TOMs) and carrier-depletion modulators (CDMs). The decoder chip includes mode-size converters (SSCs) for optical pulse input and output, a polarization splitter-rotator (PSR) for converting polarization information into on-chip path information, VOA for balancing polarization-dependent loss, MMI and TOM for polarization controller (PC). **b** Microscopic view of the encoder chip. **c** Microscopic view of the decoder chip. The encoder and decoder chip are coupled to fiber array (FA)

To validate the chip-based QDS network shown in Fig. 1, we construct a three-node quantum network to demonstrate how the QDS task operates, as illustrated in Fig. 3. This network includes a central node, Alice, acting as the signer, and two sub-nodes, Bob as the receiver and Charlie as the verifier.

Bob and Charlie each generate phase-randomized light pulses using laser diodes (LDs), with a repetition rate of 50 MHz and a pulse width of 200 ps. Bob’s and Charlie’s LDs are tuned to central wavelengths of 1549.17 nm and 1550.76 nm, respectively, which are standard in optical communication and compatible with DWDM used by Alice. Each user’s light pulses are coupled into a homemade silicon-based polarization encoder chip. A one-dimensional grating coupler (1DGC) is used for the fiber-to-chip connection. The first structure in the chip, an IM, generates signal or decoy states. It is implemented via a Mach-Zehnder interferometer (MZI), comprising two multimode interferometers (MMIs), a pair of thermal optical modulators (TOMs) providing static phase bias, and a pair of carrier depletion modulators (CDMs) for dynamic modulation.

The output of the IM is connected to a polarization modulator (Pol-M) used for polarization modulation. The Pol-M comprises an inner MZI driven by a pair of CDMs, bridging an external pair of CDMs, and finally connecting to a two-dimensional grating coupler (2DGC). The 2DGC converts the path-encoding information, modulated by the two pairs of CDMs, into polarization-encoding information and couples it into the fiber. The Pol-M can prepare the four BB84 polarization states, $\left\vert \psi \right\rangle =(\left\vert H\right\rangle +{e}^{i\theta }\left\vert V\right\rangle )/\sqrt{2}$, where *θ* ∈ {0, *π*/2, *π*, 3*π*/2}, with *θ* ∈ {0, *π*} representing the *Z* basis and *θ* ∈ {*π*/2, 3*π*/2} representing the *X* basis. The modulated polarization-encoded pulses are attenuated to the single-photon level by an off-chip variable optical attenuator (VOA) and then sent to Alice via the fiber channel.

Alice multiplexes the signal photons received from different nodes and couples them into her decoder chip using DWDM and an on-chip spot-size converter (SSC). The polarization information carried by the photons is subsequently converted into on-chip path information using a polarization splitter-rotator (PSR). The signal photons are then passively directed for measurement in the *Z* or *X* basis using two symmetric MMIs. The measurement of the photons in the *Z* and *X* basis is implemented by polarization controllers PC1 and PC2. Each polarization controller (PC) consists of a pair of TOMs and a MZI driven by another pair of TOMs. By carefully adjusting the drive voltage of the TOMs with a programmable linear DC source, PC1 and PC2 can perform measurements of the quantum state in the *Z* and *X* basis.

The photons measured by the polarization decoder chip are coupled to the external fiber through SSCs and detected by four commercial superconducting nanowire single-photon detectors (SNSPDs). These detectors have a detection efficiency of 70%, a dark count rate of approximately 30 Hz, and a dead time of 100 ns. The detection results are registered using a high-speed time-to-digital converter (TDC) and post-processed using a personal computer.

The encoder and decoder chips are realized using standard building blocks provided by a commercial fabrication foundry and are ready for large-scale production. All chips are packaged to protect them from the external environment and enable long-term operation. The size of the encoder chip is 6 × 3 mm^2^, as shown in Fig. 3b, and it is butterfly packaged with a volume of 20 × 11 × 5 mm^3^. The size of the decoder chip is 1.6 × 1.7 mm^2^, as shown in Fig. 3c, and it is packaged using a chip-in-board assembly with a size of 3.95 × 2.19 × 0.90 cm^3^.

### QDS for different fiber lengths

Using the described setup, we perform a series of QDS experiments and use the example of signing a 1 Mbit messages to demonstrate the performance of QDS. For each distance, we conduct a numerical optimization to obtain the implementation parameters to enhance the performance of key extraction between each node in the network. For example, at a distance of 150 km, Bob’s (Charlie’s) intensities of the signal and decoy states are *μ* = 0.597 (0.478) and *ν* = 0.146 (0.127), respectively. The probabilities of sending signal state *μ* and sending decoy state *ν* are set to *P*_*μ*_ = 0.808 (0.773) are *P*_*ν*_ = 0.192 (0.227), and the probability of choosing the state in *Z* (*X*) is *P*_*Z*_ = 0.947 (0.934) and *P*_*X*_ = 0.053 (0.066), respectively.

**Fig. 4:**
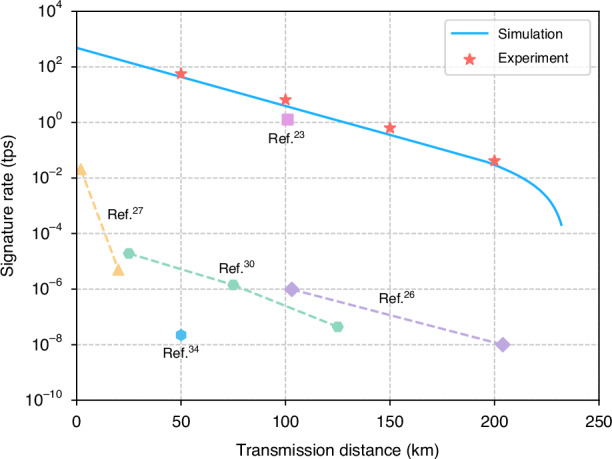
Simulation and experimental signature rates under different transmission distances. The blue solid line represents the simulated results based on the experimental parameters, while the red dots represent the experimental results at 50 km, 100 km, 150 km, and 200 km with *n*_*Z*_ = 10^7^ for finite key analysis. We also plot the highest signature rates of current QDS experiments for comparison. Note that the signature rates of our work and ref. ^23^ are for signing about 1 megabit messages, while the others are for signing 1 bit, where signing multi-bit files can only be achieved by simply repeating the process

Using the optimal implementation parameters, we successfully generate raw key bits at distances of 50 km, 100 km, 150 km and 200 km, and evaluate the signature rate conducted statistical analyses of yields and estimated average time consumption. The experimental results are plotted in Fig. 4 and detailed experimental data are provided in Supplementary Information Section 3. It can be seen that we enable to perform secure signature over different fiber spools. Particularly, we just need to use 2 × 1029-bit key to sign documents of 1 Mbit size with a security bound of 4.72 × 10^−8^ (given a signature length *L* = 1029 bits) and a signature rate up to 0.0414 tps over a fiber length of 200 km.

To demonstrate the progress entailed by our results, we compare our experimental results with current state-of-the-art QDS experiments, as shown in Fig. 4. See Table 1 for a detailed comparison. Our experiment reports the highest signature rate for QDS using the first chip-based setup. Additionally, our revised protocol achieves a higher signature rate than that reported in ref. ^23^, despite our setup has a lower repetition rate. This improvement can be attributed to lower error rates and enhanced detection efficiency obtained by using our integrated QDS system.

**Table 1:** Comparison of recent QDS experiments

References	Protocol	Clock rate	Distance	Document size	Chip	*L*	*ϵ*	*R*_*S*_
Roberts et al.^34^	MDI	1 GHz	50 km	1 bit	No	2.5 × 10^6^	10^−10^	2.22 × 10^−2^ tps
An et al.^30^	BB84	1 GHz	125 km	1 bit	No	218245	10^−10^	4.41 × 10^−2^ tps
Ding et al.^26^	BB84	50 MHz	204 km	1 bit	No	4.14 × 10^10^	10^−5^	0.01 tps
Richter et al.^27^	CV	1 GHz	20 km	1 bit	No	2.08 × 10^8^	10^−4^	5 tps
Yin et al.^23^	BB84	200 MHz	101 km	10^6^ bits	No	256	10^−32^	1.22 tps
Our work	BB84	50 MHz	100 km	10^6^ bits	Yes	783	4.64 × 10^−8^	6.50 tps
			200 km			1029	4.72 × 10^−8^	4.14 × 10^−2^ tps

*L* represents the signature length, *ϵ* is the security parameter, and *R*_*S*_ represents the signature rate

**Fig. 5:**
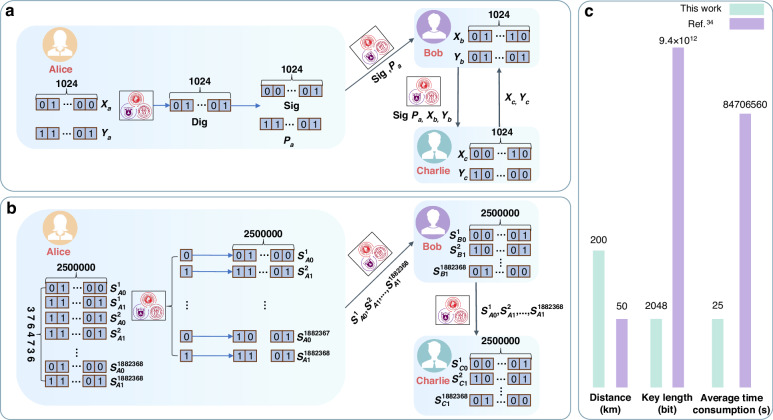
Demonstration of QDS. **a** An illustration of OTUH-QDS based on the experimental results. A picture including the school emblems of Nanjing University, Guangxi University, and Renmin University of China with a size of 1,882,368 bits (258 × 304 × 3 × 8 red green blue (RGB) color code) is signed. **b** The procedure of signing the same document as in (**a**) based on scheme in ref. ^34^. **c** Comparison of two schemes on distance, signature length and average time consumption. The green represents this work and purple represents that in ref. ^34^

To further illustrate our results, we compare the proposed scheme with the current state-of-the-art QDS system^34^ on a digital signature task for a file of approximately 1.88 M in size. We use the raw key bits collected at a distance of 200 km to perform a complete QDS process. The visual illustration is shown in Fig. 5. Our work exhibits a simple signature process capable of signing arbitrarily long documents. In contrast, the work reported in ref. ^34^ requires performing a one-bit-one signature process. Furthermore, even over longer distances (200 km vs. 50 km), our work requires only 2048 bits with an average accumulation time of 25 s to sign documents, while the work reported in ref. ^34^ requires 9.4 × 10^12^ bits with an average accumulation time of 8.4 × 10^7^ s.

## Discussion

In this work, we proposed a chip-based QDS network, where each user only needs a low-cost transmitter chip, and the measurement device is centralized at the central node to bypass the technical challenge of integrating SPD into chip. We significantly reduce the complexity of the transmitter chip and the computational cost in the post-processing stage by developing a 1-decoy-state BB84 OTUH-QDS protocol. In experimental demonstration, we achieved a higher signature rate than all previous QDS experiments by constructing a three-node QDS network using silicon photon integrated chips. To further illustrate our scheme, we conducted a signing process involving three parties using keys generated over a distance of 200 km. The results show that our scheme outperforms previous system on signature rate significantly. This work paves the way for a low-cost, wafer-scale manufactured QDS system and provides a promising scheme for integrating QDS into future quantum networks.

In the future, further research could address the challenges of our chip-based QDS network in practical applications. For example, the transmitter could be further integrated with the laser using wire bonding, or with substrates such as Indium Phosphide or hybrid integration^49,50^. This would enable the construction of a compact, chip-scale QDS transmitter for broader deployment. Furthermore, since imperfections in real-world devices, particularly in measurement devices^51^, can compromise the security of quantum systems^52,53^, it is crucial to perform a security analysis for the chip-based setup. For example, Bob and Charlie use lasers with central wavelengths of 1549.17 nm and 1550.76 nm, respectively. It is essential to assess whether Alice’s devices are compatible with both wavelengths. Additionally, the basis system in our network could be upgraded to a more secure version, such as a fully passive QKD system^54,55^. It would be valuable to explore the potential applications of this work in other important areas of quantum communication, such as quantum secure direct communication^56–63^, quantum conference key agreement^64,65^, quantum blockchain^36^, and quantum e-commerce^37^.

## Materials and methods

### Characterization of components

At a repetition rate of 50 MHz, the IM in the encoder chip held by Bob (Charlie) achieved a static extinction ratio of approximately 27 dB (25 dB) through driving TOM and a dynamic extinction ratio of about 18 dB (19 dB) through driving CDM. These parameters meet the requirements of the 1-decoy-state scheme^48^. the polarization states generated by the polarization extinction ratio of Bob’s (Charlie’s) Pol-M is approximately 23 dB (19 dB). The performance of encoder chip ensures the implementation of a low-error-rate and highly stable key generation.

To characterize the performance of the encoder chip under high-speed modulation, we measure the 3 dB bandwidth of CDM by observing eye diagrams, and the highest value reached approximately 10 GHz. This indicates our setup can support high-speed quantum state preparation with upgrading electronics control. The 3 dB bandwidth of TOM on both the encoder and decoder chips is around 3 kHz. This enables the decoder chip to provide rapid polarization tracking in field-buried and aerial fiber channel scenarios.

The 4 × 1 DWDM used is the standard version, with a 0.8 nm wavelength spacing. The insertion losses in the links from Bob to Alice and Charlie to Alice are 0.9 dB and 1.5 dB, respectively. This results in total detection efficiencies of Bob’s and Charlie’s photons of 10.3% and 9.1%, respectively. The detection efficiency includes a 7.4 dB loss from the decoder chip.

### Generalized division hash functions

In our protocol, we use generalized division hash functions to divide the input document. A generalized division hash function used in the protocol is decided by an irreducible polynomial of order *L*/8 in Galois Field (GF) (256). Note that $L/8\times {\log }_{2}256=L$, and the parameter 256 can also be set as other number. If the polynomial is *P*(*x*) and the input document is *M*, the hash function is defined as *h*(*M*) = *M*(*x*)*x*^*L*/8^ mod *P*(*x*), where all calculations are on GF(256) and *M*(*x*) is the polynomial generated by transforming every eight bits of *M* into its coefficient in turn. Note that every eight bits can be naturally mapped into an element in GF(256) through an isomorphism. The output is also a polynomial with order no more than *L*/8 − 1, and thus can be characterized into an *L*/8-element GF(256) array, and transformed into an *L*-bit string. In the demonstration we choose the *L*-bit string as the final output of the hash function, i.e., *Dig*.

It is important to note that the hash function used in our work (a generalized division hash function) differs from the one in ref. ^23^ (a linear feedback shift register Toeplitz hash function). Linear feedback shift register Toeplitz hash functions are characterized by an initial vector (an *L*-bit string) and a linear feedback shift register (another *L*-bit string), whereas the generalized division hash function is determined solely by an irreducible polynomial *P*(*x*), which is represented by an *L*-bit string. Therefore, in the distribution stage, our protocol requires only an *L*-bit string ${{\mathbb{Y}}}_{a}$, while in ref. ^23^, ${{\mathbb{Y}}}_{a}$ is a 2*L*-bit string.

### Error correction algorithms

We conducted an error correction process to obtain the identical raw keys after the key generation process. The detailed process is presented as follows:

1. The length of the key for each round of error correction is set to 10^6^. The two parties in need of error correction use prepared random sequences to shuffle the original key sequence, and set the length of each segment to 0.73/*E*_*Z*_ based on the bit error rate *E*_*Z*_^66^.
2. Each party calculates the values of parity check node of each segment and compares them with each other through a publicly authenticated channel. When differences are found, binary search is used to locate the error position. The segment with different value is divided into two equal-length blocks and the parity check codes of the two blocks are disclosed. For blocks with different parity check code values, binary search continues until the error position is located, and then one of the participants flips this bit. When a parity check code value is disclosed, the amount of information leakage increases by one.
3. In the second round, random shuffling and segmentation continue. The block length can be updated to reduce information leakage according to the new error rate. Similar to the first round, the parity check codes of each block are compared, and errors are located using binary search. Additionally, based on newly discovered error positions, errors in the first round blocks are located.
4. The above steps are repeated multiple times for the second round. Typically, two additional rounds are enough.

The error correction efficiency is always lower than 1.16 during our implementation while we set it 1.16 during simulation.

## Supplementary information

Supplementary Information for Chip-integrated quantum signature network over 200 km

## Data Availability

The data that support the results of this work are available from the corresponding author on reasonable request.
