# Supplementary material for: Chip-integrated quantum signature network over 200 km
Source: Light Sci Appl. 2025 Mar 4;14:108. doi: 10.1038/s41377-025-01775-4 (PMC11880301; doi:10.1038/s41377-025-01775-4)
Supplement: Supplementary file 1 — Supplementary Information for Chip-integrated quantum signature network over 200 km [file 41377_2025_1775_MOESM1_ESM.pdf]

Supplementary Information for Chip-integrated quantum signature network over 200 km

Yongqiang Du[1,7], Bing-Hong Li[2,7], Xin Hua[3,7], Xiao-Yu Cao[2,7], Zhengeng Zhao[1], Feng Xie[1,4], Zhenrong Zhang[4], Hua-Lei Yin[2,5,*], Xi Xiao[3,6,*], Kejin Wei[1,*]

[1]*Guangxi Key Laboratory for Relativistic Astrophysics, School of Physical Science and Technology, Guangxi University, Nanning 530004, China.*
[2]*National Laboratory of Solid State Microstructures and School of Physics, Collaborative Innovation Center of Advanced Microstructures, Nanjing University, Nanjing 210093, China.*
[3]*National Information Optoelectronics Innovation Center (NOEIC), Wuhan 430074, China.*
[4]*Guangxi Key Laboratory of Multimedia Communications and Network Technology, School of Computer, Electronics, and Information, Guangxi University, Nanning 530004, China.*
[5]*Department of Physics and Beijing Key Laboratory of Opto-electronic Functional Materials and Micro-nano Devices, Key Laboratory of Quantum State Construction and Manipulation (Ministry of Education), Renmin University of China, Beijing 100872, China.*
[6]*Peng Cheng Laboratory, Shenzhen 518055, China.*
[7]*These authors contributed equally to this paper.*
[*]*hlyin@ruc.edu.cn*
[*]*xxiao@wri.com.cn*
[*]*kjwei@gxu.edu.cn*

Section 1: 1-decoy-state OTUH-QDS protocol

Here, we provide a detailed explanation of the specific steps involved in the distribution stage and the messaging stage of the 1-decoy-state OTUH-QDS protocol.

Distribution stage. Bob and Charlie will independently implement the 1-decoy-state BB84 key generation protocol (KGP) with Alice to distribute a pair of identical raw bit strings. The specifics are as follows:

1. Transmission. Bob (Charlie) randomly selects a bit value, chooses an intensity choice $k \in \{\mu, \nu\}$ ({ signal, decoy }) with probabilities $P_k \in \{P_\mu, P_\nu\}$, and selects a basis $\lambda \in \{Z, X\}$ with probabilities $P_\lambda \in \{P_Z, P_X\}$. Based on these selections, Bob (Charlie) prepares a phase-randomized weak coherent pulse from four states $\{|0_Z\rangle, |1_Z\rangle, |0_X\rangle, |1_X\rangle\}$, where $\{|i_\lambda\rangle\}$ represents the state corresponding to bit "i" in the "λ" basis. Finally, Bob (Charlie) sends this prepared state to Alice via the quantum channel.

2. Detection. Alice selects a basis from $\{Z, X\}$ with probabilities $\{P_Z, P_X\}$ to measure the received pulses. For events resulting in a detection click, she records both the basis choice and the corresponding measured bit value. (For double-click events, she assigns a bit value at random.)

3. Basis reconciliation. Bob (Charlie) and Alice publicly announce their basis and intensity choices via an authenticated channel. Then, they determine the number of detection events $n_{\lambda,k}$, when both Bob (Charlie) and Alice use the basis λ for intensity k .

4. Parameter estimation. Bob (Charlie) and Alice announce the bit information of their detection events measured under the X basis. Then, they determine the number of error pulses $m_{X,k}$, where both Bob (Charlie) and Alice, under intensity k, utilize the X basis and obtain inconsistent bit values. Subsequently, they use the 1-decoy-state method[1] to estimate the lower bounds of vacuum events $s^l_{Z,0}$ and single-photon events $s^l_{Z,1}$, as well as the upper bound of the phase error rate of single-photon events ϕ^u_Z associated with the Z basis.

5. Error correction and verification. Bob (Charlie) and Alice utilize the bit information measured in the Z basis to generate the raw key. They reveal λ_{EC} bits of information to perform an error correction step capable of correcting errors for the expected quantum bit error rate (QBER) E_Z. To ensure that both parties share a pair of identical keys with a correctness of ε_{cor}, they execute an error verification step using two universal hash functions, which publish $\lceil \log_2 1/\varepsilon_{\text{cor}} \rceil$ bits of information.

By executing steps 1-5, Bob (Charlie) and Alice distribute a pair of identical raw keys of size n_Z, denoted as K_b (K_c), respectively. Subsequently, Alice generates a new key string $K_a = K_b \oplus K_c$ by performing an XOR operation on her key strings K_b and K_c. If the signing of a message M requires $2L$ bits of the key, Alice randomly distills $2L$ key bits from her key string K_a to create two L-bit key strings $\{\mathbb{X}_a, \mathbb{Y}_a\}$. She then announces the positions of these bits to Bob and Charlie via an authenticated channel. Upon receiving this information, Bob and Charlie extract their corresponding key strings $\{\mathbb{X}_b, \mathbb{Y}_b\}$ and $\{\mathbb{X}_c, \mathbb{Y}_c\}$ from K_b and K_c at the positions indicated by Alice, ensuring that

the relationships $\mathbb{X}_a = \mathbb{X}_b \oplus \mathbb{X}_c$ and $\mathbb{Y}_a = \mathbb{Y}_b \oplus \mathbb{Y}_c$ are satisfied. The remaining portions of the key strings K_a, K_b, and K_c can be re-randomly extracted for subsequent signing tasks until they are fully utilized.

Messaging stage. Upon completion of the distribution stage, Alice, Bob, and Charlie can proceed to sign the message M at any time using the following steps:

1. Signing of Alice. Alice randomly generates an irreducible polynomial in a Galois field using a local quantum random number generator, which can be denoted as an L-bit string P. Then she constructs a generalized division hash function $H_P(x)$ based on P. Subsequently, she inputs the message to be signed (denoted as M) into $H_P(x)$ to derive the digest $Dig = H_P(M)$. Then, she encrypts the digest using the key string $\mathbb{X}_a$ to obtain the signature $Sig = Dig \oplus \mathbb{X}_a$, while also encrypting the random bit string P using $\mathbb{Y}_a$ to obtain $P_a = P \oplus \mathbb{Y}_a$. Subsequently, she transmits $\{Sig, M, P_a\}$ through an authenticated channel to Bob.

2. Transference. Bob utilizes an authenticated classical channel to transmit the received $\{Sig, M, P_a\}$ along with his key bit strings $\{\mathbb{X}_b, \mathbb{Y}_b\}$ to Charlie. Subsequently, Charlie also employs an authenticated channel to send his key bit strings $\{\mathbb{X}_c, \mathbb{Y}_c\}$ to Bob.

3. Verification. Bob and Charlie will independently verify the signature. To accomplish this, Bob creates two new key strings $\{K_{\mathbb{X}_b} = \mathbb{X}_b \oplus \mathbb{X}_c$, $K_{\mathbb{Y}_b} = \mathbb{Y}_b \oplus \mathbb{Y}_c\}$ through XOR operations. Then, he uses $K_{\mathbb{X}_b}$ and $K_{\mathbb{Y}_b}$ to decrypt the expected digest $Dig_b^{'} = Sig \oplus K_{\mathbb{X}_b}$ and the random sequence $P_b = P_a \oplus K_{\mathbb{Y}_b}$ via XOR operations, respectively. Following this, he constructs a hash function using P_b and applies the hash operation to obtain the actual digest Dig_b of the message M . If the actual digest is equal to the expected digest, Bob will accept the signature; otherwise, he will reject the signature and abort the protocol.

If Bob accepts the signature, Charlie will also perform similar steps for verification. She generates a new key bit string $\{K_{\mathbb{X}_c} = \mathbb{X}_b \oplus \mathbb{X}_c$, $K_{\mathbb{Y}_c} = \mathbb{Y}_b \oplus \mathbb{Y}_c\}$ using Bob's key and his own key, and uses this new key string to decrypt the expected digest $Dig_c^{'} = Sig \oplus K_{\mathbb{X}_c}$ and the random sequence $P_c = P_a \oplus K_{\mathbb{Y}_c}$ through XOR operations. Then, he constructs a hash function using P_c and applies the hash operation to obtain the actual digest Dig_c of M. The signature is accepted if the two digests are the same; otherwise, the signature is rejected.

Section 2: Security analysis of OUTH-QDS

Here, we will analyze the security parameters of our protocol resisting robustness, forgery, and non-repudiation. In this protocol, we use imperfect keys with privacy leakage to encrypt the hash value and hash function. Thus, any possible attackers may obtain partial information on the keys. In the finite-size scenario, Alice, Bob, and Charlie generate the key string $K \in \{\mathbb{X}_a, \mathbb{Y}_a, \mathbb{X}_b, \mathbb{Y}_b, \mathbb{X}_c, \mathbb{Y}_c\}$ using KGP. E as an eavesdropper's system after performing optimal measurement strategy on K. According to the decoy-state method[1,2], the min-entropy of K and E is

$$H_{\text{min}}(K|E) \leq s_{Z,0}^L + s_{Z,1}^L \left(1 - h\left(\phi_Z^{L,u}\right)\right) - \frac{L}{n_Z}\left(\lambda_{\text{EC}} + \log_2\left(2/\epsilon_{\text{cor}}\right)\right), \tag{S1}$$

where $s_{Z,0}^L$ is the lower bound of the vacuum state event that Alice detects Bob (Charlie) sending under the Z basis in an L-bit string, $s_{Z,1}^L$ is the lower bound of the single-photon state event that Alice detects Bob (Charlie) sending under the Z basis in an L-bit string, $\phi_Z^{L,u}$ is the upper bound of the phase error rate of the single-photon state event that Alice detects Bob (Charlie) sending under the Z basis in an L-bit string. λ_{EC} is the number of bits revealed in the error correction process. $\epsilon_{\text{cor}} = \varepsilon_{\text{hash}}$ is the parameter for evaluating correctness. The $h(x) = -x\log_2 x - (1-x)\log_2(1-x)$ is the binary Shannon entropy function.

Furthermore, we will demonstrate how to derive the different parameters in Eq. S1 from experimental data. To visually depict this process, we partition the parameter estimation into two segments, as shown in Fig. S1. In the protocol, Alice first shares an n_Z-bit string K_b with Bob and K_c with charlie, and generates $K_a = K_b \oplus K_c$ as her own key string. Three parties then disturb the order of their strings and divide them into L-bit sub-strings. Parameter $s_{Z,0}^L$, $s_{Z,1}^L$, $\phi_Z^{L,u}$ is about an L-bit sub-string and can be estimated from parameters that characterize the n_Z-bit string.

For events n_Z measured in the Z basis during the distribution process, the lower bounds of vacuum events and single-photon events are estimated as follows[1]:

$$s_{Z,0} \geq s_{Z,0}^l := \frac{\tau_0}{\mu - \nu}\left(\mu n_{Z,\nu}^- - \nu n_{Z,\mu}^+\right), \tag{S2}$$

$$s_{Z,1} \geq s_{Z,1}^l := \frac{\tau_1 \mu}{\nu(\mu - \nu)}\left(n_{Z,\nu}^- - \frac{\nu^2}{\mu^2} n_{Z,\mu}^+ - \frac{\left(\mu^2 - \nu^2\right)}{\mu^2}\frac{s_{Z,0}^u}{\tau_0}\right), \tag{S3}$$

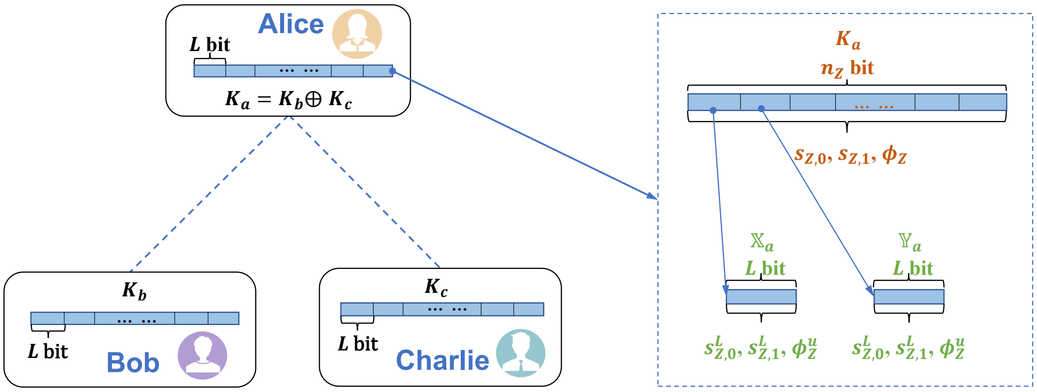

Figure S1: An illustration of finite key analysis and corresponding parameters. In the distribution stage each of Alice, Bob, and Charlie holds an n_Z-bit key string that will later be divided into L-bit sub-strings that are used for generating the signature. The n_Z-bit key string contains $s_{Z,0}$ vacuum events and $s_{Z,1}$ single-photon events, and the phase error rate is ϕ_Z. For an L-bit sub-string, it contains $s^L_{Z,0}$ vacuum events and $s^L_{Z,1}$ single-photon events, and the upper bound of phase error rate is ϕ^u_Z.

where $\tau_n := \sum_{k\in\mathcal{K}} P_k e^{-k}k^n/n!$ represents the total probability of sending an n-photon state, and $n^{\pm}_{Z,k}$ denotes the correction for the observed values in intensity $k \in \{\mu, \nu\}$ due to statistical fluctuations considered through the Hoeffding inequality[3], given by

$$n^{\pm}_{Z,k} := \frac{e^k}{P_k}\left(n_{Z,k} \pm \sqrt{\frac{n_Z}{2}\log\frac{1}{\varepsilon_{SF}}}\right), \tag{S4}$$

and the upper bound of vacuum events under finite data size is given by

$$s_{Z,0} \le s^u_{Z,0} := 2\left(\tau_0\frac{e^k}{P_k}\left(m_{Z,k} + \sqrt{\frac{m_Z}{2}\log\frac{1}{\varepsilon_{SF}}}\right) + \sqrt{\frac{n_Z}{2}\log\frac{1}{\varepsilon_{SF}}}\right), \tag{S5}$$

$m_{Z,k}$ represents the total number of error events in intensity k. $n_Z = \sum_{k\in\mathcal{K}} n_{Z,k}$ and $m_Z = \sum_{k\in\mathcal{K}} m_{Z,k}$ respectively represent the total number of events and the number of error events in the Z basis. ε_{SF} represents the failure probability of statistic fluctuation that is set as 10^{-10}.

For events measured in the X basis, we utilize the following formula to estimate the phase error rate of the single-photon events in the Z basis

$$\phi_Z := \frac{c_{Z,1}}{s_{Z,1}} \le \frac{v_{X,1}}{s_{X,1}} + \gamma^U\left(s_{Z,1}, s_{X,1}, \varepsilon_{SF}, \frac{v_{X,1}}{s_{X,1}}\right), \tag{S6}$$

where

$$\gamma^U(n,k,\epsilon,\lambda) = \frac{\frac{(1-2\lambda)AG}{n+k} + \sqrt{\frac{A^2G^2}{(n+k)^2} + 4\lambda(1-\lambda)G}}{2 + 2\frac{A^2G}{(n+k)^2}}, \tag{S7}$$

in which

$$A = \max\{n,k\}, \tag{S8}$$

and

$$G = \frac{n+k}{nk}\ln\left(\frac{n+k}{2\pi nk\lambda(1-\lambda)\epsilon^2}\right). \tag{S9}$$

The upper bound on the number of error bits for single-photon events in the X basis can be estimated using the following formula

$$v_{X,1} \le v^u_{X,1} = \frac{\tau_1}{\mu-\nu}(m^+_{X,\mu} - m^-_{X,\nu}), \tag{S10}$$

$m^{\pm}_{X,k}$ represents the observed number of errors bits for intensity k considering a scenario with finite key, and it can be expressed as

$$m^{\pm}_{X,k} := \frac{e^k}{P_k}\left(m_{X,k} \pm \sqrt{\frac{m_X}{2}\log\frac{1}{\varepsilon_{SF}}}\right). \tag{S11}$$

We can now compute the upper bound on the phase error rate of single-photon events in the Z basis using the following formula

$$\phi_Z \leq \phi_Z^u := \frac{v_{X,1}^u}{s_{X,1}^l} + \gamma^U \left(s_{Z,1}^l, s_{X,1}^l, \varepsilon_{SF}, \frac{v_{X,1}^u}{s_{X,1}^l} \right). \tag{S12}$$

The estimation method of the lower bound $s_{X,1}^l$ for single-photon events in the X basis is similar to the analysis of $s_{Z,1}^l$ in the Z basis.

In the protocol Alice, Bob, and Charlie all randomly divide their keys into L-bit strings. This is a random sampling without replacement process, and thus the statistical fluctuation can be bounded by γ^U. Then we can obtain the bound of $s_{Z,0}^L$, $s_{Z,1}^L$, and ϕ_Z^u in Eq. S1

$$s_{Z,0}^L \geq L \left[s_{Z,0}/n_Z - \gamma^U (L, n_Z - L, \varepsilon_{SF}, s_{Z,0}/n_Z) \right], \tag{S13}$$

$$s_{Z,1}^L \geq L \left[s_{Z,1}/n_Z - \gamma^U (L, n_Z - L, \varepsilon_{SF}, s_{Z,1}/n_Z) \right], \tag{S14}$$

$$\phi_Z^{L,u} \leq \phi_Z^u + \gamma^U \left(s_{Z,1}^L, s_{Z,1}^l - s_{Z,1}^L, \varepsilon_{SF}, \phi_{Z,1} \right). \tag{S15}$$

The amount of information the attacker obtain can be estimated by Eq. S1, and the maximum probability that an attacker successfully guesses one of the strings X_b, Y_b, X_c, Y_c is no more than

$$Pr = 2^{-H_n}, \tag{S16}$$

where $H_n = H_{\text{min}}(K|E)$ in Eq. S1, represents the unknown information to the eavesdropper within the n-bit string generated in the distribution stage.

Additionally, there are three security parameters in the QDS process that require analysis.

Robustness The honest run aborting will occur only when Alice and Bob (or Charlie) share different key bits after the distribution stage. In our protocol, Alice and Bob (Charlie) will perform error correction in distribution stage. Thus, they will share the identical final key, unless the error correction fails. The robustness bound is $\epsilon_{\text{rob}} = 2\epsilon_{\text{cor}}$.

Repudiation For Alice's repudiation attacks, Bob and Charlie are both honest and symmetric and they have the same new key strings. They will make the same decision for the same document and signature. In other words, when Bob rejects (accepts) the document, Charlie also rejects (accepts) it. Therefore, our QDS protocol is immune to repudiation naturally, i.e., the repudiation bound $\epsilon_{\text{rep}} = 0$.

Forgery Bob forges successfully when Charlie accepts the forged document forwarded by Bob. According to our protocol, Charlie accepts the message if and only if Charlie gets the same result through one-time pad encryption and one-time hash functions. Since the signature is encrypted by one-time pad and each hash function is utilized only one time, in a specific round Bob can obtain no information about the hash function or the hash value from previous rounds. Therefore, the probability of a successful forgery is identical to the failure probability of authentication scheme based on hashing, i.e., one finds two distinct documents to have the identical hash value[4]. Due to the construction of hash functions, Bob's optimal strategy is to guess the monic irreducible polynomial $p(x)$ which can be concluded if successfully guessing X_c. Also, since he knows that $p(x)$ is irreducible, he can guess only from all irreducible polynomials (no less than $2^{(n+2)}/n$) rather than all polynomials (2^n). The probability that Bob successfully guesses $p(x)$ is $n/4Pr$. To finish a forgery attack, Bob can generate a polynomial $g(x)$ of order no more than m, and construct $M'(x) = M(x) + g(x), Sig' = Sig$. As long as $p(x)$ is a factor of $g(x)$, Charlie will pass M', Sig'. Thus, Bob can guess $p(x)$ for no more than $m/8$ times. The forgery bound is $\epsilon_{\text{for}} = \frac{m}{8 \cdot 2^{H_n - 1}}$.

The security bound of the QDS protocol, i.e., the maximum probability that an attack is successfully performed, is defined as $\epsilon = \max\{\epsilon_{\text{rob}}, \epsilon_{\text{rep}}, \epsilon_{\text{for}}\}$.

It can be seen that ϵ_{rob} and ϵ_{rep} are fixed numbers, and only ϵ_{for} is decided by H_n influenced by parameters such as channel loss and signature length L. Thus, the variation of ϵ is mainly caused from ϵ_{for}. In other words, the selection of L is constrained by ensuring ϵ_{for} being small enough.

Section 3: Detailed experimental results

Table 1 shows the detailed experimental results.

Table 1: Experimental parameters and results on the Bob (Charlie)-Alice link. A, B, and C represent users Alice, Bob, and Charlie, respectively. T and Loss represent the transmission distance and loss of the Bob (Charlie)-Alice fiber link, respectively. μ (ν) is the intensity of the signal (decoy) state sent by Bob and Charlie, and P_μ (P_ν) represents the probability of selecting intensity μ (ν). P_Z (P_X) represents the probability of Bob and Charlie selecting the Z (X) basis. $n_{Z,\mu}$ ($n_{Z,\nu}$) represents the total number of detection events when Bob and Charlie send the signal (decoy) state in the Z basis and Alice measures in the Z basis. $m_{Z,\mu}$ ($m_{Z,\nu}$) represents the total number of detection errors when Bob and Charlie send the signal (decoy) state in the Z basis and Alice measures in the Z basis. $m_{X,\mu}$ ($m_{X,\nu}$) represents the total number of detection errors when Bob and Charlie send the signal (decoy) state in the X basis and Alice measures in the X basis. n_Z represents the total accumulated detection events in the Z basis after basis reconciliation, t represents the time required to accumulate the data size of n_Z, $s^l_{Z,1}$ represents the lower bound of the single-photon detection events in the Z basis, E_Z represents the quantum bit error rate in the Z basis, ϕ^u_Z represents the upper bound of the phase error rate in the Z basis, λ_{EC} represents the number of bits leaked in the error correction step, L represents the signature length, ϵ is the security parameter, and R_S represents the optimal signature rate achievable at each link. Note that a QDS process requires two links and the actual signature rate is the low one of the two rates.

T (km)	Link	Loss (dB)	μ	ν	P_μ	P_ν	P_Z	P_X	$n_{Z,\mu}$	$m_{Z,\mu}$	$n_{X,\mu}$	$m_{X,\mu}$	$n_{Z,\nu}$
50	A-B	9.72	0.601	0.147	0.807	0.193	0.947	0.053	9449854	51823	516479	2387	550146
	A-C	9.72	0.481	0.127	0.775	0.225	0.935	0.065	9280871	130700	561044	8154	719129
100	A-B	19.24	0.599	0.147	0.807	0.193	0.948	0.052	9448344	54494	511670	3523	551656
	A-C	19.24	0.479	0.127	0.775	0.225	0.935	0.065	9279475	119659	597173	5052	720525
150	A-B	29.16	0.597	0.146	0.808	0.192	0.947	0.053	9449307	63862	525201	1935	550693
	A-C	29.16	0.478	0.127	0.773	0.227	0.934	0.066	9279885	95510	598243	8926	720115
200	A-B	39.23	0.593	0.144	0.798	0.202	0.935	0.065	9411383	92289	638273	6601	588617
	A-C	39.23	0.472	0.123	0.768	0.232	0.918	0.082	9240824	186323	810542	14213	759176

T (km)	Link	$m_{Z,\nu}$	$n_{X,\nu}$	$m_{X,\nu}$	n_Z	t (s)	$s^l_{Z,1}$	E_Z	ϕ^u_Z	λ_{EC}	L	ϵ	R_S (tps)
50	A-B	4781	27760	251	10^7	74.8	4878658	0.566%	0.0202	581997	698	4.65×10^{-8}	90.8
	A-C	12469	44603	1069	10^7	105.4	5605116	1.432%	0.0353	1188112	844	4.56×10^{-8}	56.2
100	A-B	6631	28700	324	10^7	662.6	4849083	0.611%	0.0270	595119	735	4.78×10^{-8}	10.3
	A-C	13943	39847	536	10^7	981.7	5642925	1.336%	0.0312	1136046	783	4.64×10^{-8}	6.50
150	A-B	7326	28411	224	10^7	6686.3	4872943	0.712%	0.0156	681965	718	4.61×10^{-8}	1.04
	A-C	13063	44413	984	10^7	9775.2	5559470	1.086%	0.0436	967145	812	4.78×10^{-8}	0.630
200	A-B	18264	40011	1268	10^7	79799.4	4736584	1.106%	0.0254	965191	922	4.77×10^{-8}	6.46×10^{-2}
	A-C	31614	72330	3316	10^7	117291.2	5567378	2.179%	0.0251	1726908	1029	4.72×10^{-8}	4.14×10^{-2}

Note: The detection efficiency is 60% at 200 km and 70% at other distances.

References

[1] Rusca, D. et al. Finite-key analysis for the 1-decoy state QKD protocol. *Appl. Phys. Lett.* **112**, 171104 (2018).

[2] Lim, C. C. W. et al. Concise security bounds for practical decoy-state quantum key distribution. *Phys. Rev. A* **89**, 022307 (2014).

[3] Hoeffding, W. *Probability Inequalities for sums of Bounded Random Variables*, 409–426 (Springer New York, New York, NY, 1994).

[4] Li, B. H. et al. One-time universal hashing quantum digital signatures without perfect keys. *Phys. Rev. Appl.* **20**, 044011 (2023).
